# Adaptation of the Diet Quality Questionnaire as a Global Public Good for Use in 140 Countries^☆^

**DOI:** 10.1016/j.cdnut.2024.104499

**Published:** 2024-11-02

**Authors:** Anna W Herforth, Kristina Sokourenko, B Cecilia Gonzalez, Betül TM Uyar, Andrea LS Bulungu, Chris Vogliano

**Affiliations:** 1 Department of Global Health and Population, Harvard T.H. Chan School of Public Health, Boston, MA, United States; 2 Division of Human Nutrition and Health, Wageningen University and Research, Wageningen, Netherlands; 3 Department of Global Development, Cornell University, Ithaca, NY, United States; 4 Independent consultant, Baltimore, MD, United States; 5 Brown School, Washington University in St. Louis, St. Louis, MO, United States

**Keywords:** DQQ, minimum dietary diversity, MDD, MDD-C, MDD-W, dietary assessment, nutritional anthropology, nutrition surveillance

## Abstract

**Background:**

Food group consumption data are useful for measuring and monitoring diet quality. To collect valid data across contexts, consistent and rigorous adaptation of survey questions is needed.

**Objectives:**

The objective of this research was to adapt food group consumption survey questions for 140 countries, by identifying the most common (sentinel) food items in each food group using a structured, participatory process and global standards for classification.

**Methods:**

Survey questions were adapted for 29 food groups of the Diet Quality Questionnaire (DQQ) and for additional questions for infant and young child feeding (IYCF) indicators. For each country, adaptation comprised the following: *1*) review of existing questionnaires, dietary intake data, and other information to draft food lists; *2*) key informant (KI) interviews with 5–12 experts to identify and prioritize sentinel items including terminology; *3*) comparison of items across countries within the same region to identify inconsistencies, and follow-up with KIs to resolve them.

**Results:**

In total, 1016 KIs contributed to the adapted DQQs for 140 countries and IYCF DQQs for 96 countries, amounting to ∼9550 h of collective effort (68 person-hours/country on average) from 2020 to 2024. The process revealed numerous challenges and decisions to ensure consistent classification of items and valid question formulation.

**Conclusions:**

Country-specific questions adhering to global standards, and adapted through cumulative and iterative input of local experts, enable the collection of food group consumption data that are valid and comparable across time and geographies. The adapted survey questions have been implemented in the Demographic and Health Surveys and Gallup World Poll in 94 countries, generating the first cross-country data on Minimum Dietary Diversity and other diet quality indicators. The finalized country-adapted DQQs and IYCF DQQs were translated to 143 national languages and are published online as a global public good for population-level diet quality measurement.

## Introduction

Diet quality monitoring is needed to provide vital information about the causes of poor health and malnutrition and outcomes of food systems. Monitoring of diet quality has been hindered by a lack of feasible, valid data collection tools and consequent gaps in data [1]. The measurement of consumption of food groups, rather than food items and quantities of food items, can greatly simplify data collection, reducing costs and expertise requirements and making diet quality monitoring feasible at scale. Several indicators based on food group consumption in the previous day have been developed for diet quality monitoring at the population level. These include minimum dietary diversity (MDD) as a proxy of nutrient adequacy for women aged 15–49 y (MDD-W) and for children aged 6-23 mo (MDD-C), other infant and young child feeding (IYCF) indicators for children aged 6–23 mo, and indicators related to risk of NCDs for the general population [[2], [3], [4]]. These food-group based indicators have been incorporated into regional and global monitoring frameworks, including MDD as a proposed indicator in the Sustainable Development Goals [[5], [6], [7]].

There are several methods for obtaining food group consumption data. One is deriving them from existing nationally representative dietary intake surveys. That type of data are not available in most countries [8]. A second method is open recall, where enumerators ask respondents to list everything they ate or drank the previous day and record each item. Enumerators must receive specific training in how to probe for items often forgotten, and survey teams must have detailed knowledge of local foods and food names, as well as global food group classification systems, to categorize food items correctly. A third method is list-based food group questions, in which respondents are asked yes or no questions on whether they have consumed any item on a list of foods in the previous day. Research has shown that open recall and list-based methods are comparably accurate and that the list-based method costs less and is simpler to implement, requiring much less enumerator training [9,10]. To achieve accurate results, however, list-based questions need to be well adapted.

A problem with the list-based approach is that adaptation is difficult to do well, and each user has had to adapt the questions independently. As a result, different adaptations are not necessarily comparable, may not follow standardized global guidance, and many duplicative efforts have taken place. Each survey team is likely to produce a different adaptation for the same context, varying based on the skills, knowledge, time and resources available for adaptation. The expertise and effort required for question adaptation is sometimes a prohibitive barrier to data collection. Moreover, researchers using list-based questionnaires rarely publish their survey tools or describe their methods for local adaptation. Thus, the cognitive validity, rigor, and consistency of each adaptation are generally unexamined, and the validity of survey results cannot be assessed.

Major problems in adaptation are: *1*) misclassifying items, *2*) omitting items that are common, *3*) including items that should be skipped because they are typically consumed in small amounts (<15 g), duplicative, extraneous or rarely consumed, *4*) using terminology that is technically correct but not understood by everyone (e.g. legumes), and *5*) creating questions that lack cognitive validity because they are too long or confusing. An example of a questionnaire of typical quality, adapted by a local survey team, is shown in Supplemental Table 1; all but 2 questions (cheese and eggs) suffered from 1 or more of the above adaptation problems. For data used for global comparisons, not only do the indicators need to be the same, but also the underlying data need to be collected comparably. If list-based questionnaires were developed for each country in isolation, the chances would be high of inconsistent inclusion, exclusion, and categorization of food items, as well as inconsistent cognitive validity of questions.

The Minimum Dietary Diversity for women aged 15–49 y (MDD-W) updated guide for measurement (2021) cites the extensive preparation of survey tools as a disadvantage to the list-based method, noting, “substantial up-front investment is required to develop food lists for a given context or country…Once the preparatory work has been completed in a specific geographic area, subsequent surveys can use the same adapted food lists, enumerator instructions and guidance sheets, which will greatly reduce preparation time.” (p49) [11]. Best practices for the list-based approach have become clearer: the first MDD-W Guide to Measurement (2016) suggested an open-ended question approach for collecting food group consumption data, with question such as, “Any fruits that are dark yellow or orange inside, like ripe mango, ripe papaya, [other local vitamin A-rich fruits]” [12]. This type of question requires respondents to think of other foods that might belong in the category and categorize them in the same way researchers intend. Results reported by Herforth et al. [13], Namaste et al. [14], and Khadka et al. [15] show that respondents frequently misclassify food items when they are asked to identify foods that belong in the category; for example, in response to the abovementioned question oranges may be reported, which are not rich in vitamin A and do not belong in the category. Accordingly, FAO updated the guidance for the list-based approach to adopt closed-ended questions composed of sentinel foods [11]. Sentinel foods are the items that capture the vast majority of people consuming a food group in a given context.

The objective of this research was to adapt list-based survey questions to capture food group consumption for every low-income and middle-income country covered by international surveys. We aimed to identify the most common (sentinel) food items in each food group using a structured, participatory process and global standards for classification. The adapted questions were needed for implementation in both the Demographic and Health Surveys (DHS), implemented in ≤93 countries every 5 y, and the Gallup World Poll (GWP), implemented in >140 countries annually. The DHS-8 core questionnaire included MDD-W for the first time starting in 2020, requiring new country-adapted questionnaires; this process also presented an opportunity to revisit country adaptations of existing IYCF questionnaires, aligned with the woman’s questionnaire. This study therefore sought to provide consistent, rigorous adapted questions for adults and infants and young children (IYC), so that comparable data would be collected across the life course in DHS and so that comparable data would be collected between the GWP and DHS. Ready and comparable data collection instruments are also of interest to governments, which implement other national household surveys such as the Living Standards Measurement Study (LSMS) and programmatic surveys such as those implemented by Feed the Future [16]. Standard country-adapted questionnaires would enable data collection by any survey team, obviate duplicative adaptation efforts, and improve the consistency and validity of data collection for MDD and other diet quality indicators.

## Methods

Survey questions for the general population were adapted for 29 food groups of the Diet Quality Questionnaire (DQQ), designed to capture the food groups required for calculating the MDD-W and additional indicators related to NCDs [17]. Survey questions for IYC were adapted for the 29 DQQ food groups, as well as additional questions on breastmilk substitutes, milk, yogurt, and other food groups (insects, organ meats, and red palm oil) following the model questionnaire published by WHO and UNICEF [4]. The same food groups are used in all settings, but the food items are adapted for each country using a closed-ended question approach [11,17].

Food items were categorized according to the classification criteria in the FAO MDD-W updated guide for measurement and additional DQQ food group definitions [11,18] (Supplemental Table 2). A classification system for whole grain foods was developed based on USDA criteria [19]. Whole grain foods were defined as those made from entire cereal grain seeds, including barley, oats, sorghum, fonio, millet, teff, whole wheat, whole grain maize, or brown/red/black rice. When an item is made from a mix of whole and refined grains, it is counted as whole grain if whole grains account for at least half of the mix; if food composition information was available, fiber content of 4 g or more per 100 g served as a marker that the item was likely whole grain. An item is generally excluded from the whole grain category when it can be made with either refined or whole grain dependent upon individual recipe or preference or when respondents cannot reliably distinguish between refined and whole grain products (e.g. bread) [20]. Where an item is a whole grain as consumed by the majority of the population however (e.g. chapati in India, most commonly made with whole wheat or *atta* flour), it is kept in the whole grain category.

The adaptation of the DQQ and IYCF DQQ questions for each country comprised 3 main steps [11]. First, a desk review was conducted to draft a preliminary food list for each food group. Second, key informant interviews (KIIs) were conducted with individuals having knowledge and expertise in the local food system to identify select and prioritize the most commonly consumed foods in each food group (sentinel foods) and identify the common names by which they are known. The third step was a harmonization process, where the sentinel foods identified in each food group in each country were compared with foods independently identified in neighboring countries. Where there were unexplained discrepancies, key informants (KIs) were recontacted to provide clarifying information about the items in question, and the food lists were revised accordingly. The adaptation team was composed of the authors with qualitative research skills and subject matter expertise in food and nutrition and in other relevant fields including agriculture, survey research, and anthropology. The 3 main adaptation steps are detailed in the following sections.

### Creating initial draft food lists

For each country, preliminary food lists were drafted from existing written resources. These included dietary intake data, questionnaires, food lists from consumer price monitoring or household consumption and expenditure surveys, and other publications about common crops and foods. Other sources to inform initial drafts included food composition tables developed for the region, academic articles and books [[21], [22], [23]], culinary blogs, and personal experience of the adaptation team living or working in a particular country. As adaptations for more countries became completed, the survey team referred to adaptations in neighboring countries to create initial drafts for new countries, because similar foods are often common across political borders, where elements of culture and ecology are shared. Different countries had different levels of information available as a starting point. Except in rare cases where national dietary intake data were available and analyzed in parallel studies (Brazil, China, Ethiopia, Lao PDR, Mexico, Switzerland, and United States) [3,8,24,25], existing resources were suggestive of potentially common items rather than definitive. The initial drafts provided a basis for discussion and feedback from KIs by illustrating the types of foods that belonged in the food group, rather than starting from a blank page.

### Key informant interviews

KIIs were the primary source of information for adaptation. KIs in each country were identified who were familiar with commonly consumed foods, food culture, and how foods are called. The adaptation team sought out KIs with different areas of expertise and from different sociodemographic groups and geographic locations within each country. Often, KIs were based in urban areas, but we sought KIs who had come from or currently work with rural communities. Several international organizations (FAO, WHO, World Food Programme (WFP), Scaling Up Nutrition Secretariat, and International Union of Nutrition Scientists) provided letters of support for the adaptation endeavor, and staff from these organizations also helped to identify KIs knowledgeable about food and diets in each country. These contacts and written letters often improved the response rates and involvement of KIs connected to each respective organization. Where direct contacts were insufficient, a snowball process identified additional KIs.

KIIs were held virtually via videoconference on Zoom, Google Meet, Teams, Skype, or WhatsApp. A small number of interviews were conducted in person or completed through email in rare cases of insufficient network connectivity. KIIs were mostly conducted one-on-one but were sometimes conducted in small groups of 2 or 3 where multiple people from the same organization served as KIs. Interviews lasted 60–120 min, held in 1 or 2 sessions. KIIs were conducted in English, French, Spanish, Portuguese, Russian, Armenian, and Turkish.

The focus of the adaptation was identifying sentinel foods for each food group in each country using the names by which the foods are known. For each of the 29 food groups, each KI was asked to help identify the most commonly consumed foods, including both the common name for the item and its name or description in English or Latin. KIs with specific expertise on IYCF were sought to adapt IYCF questions, requiring familiarity with infant formula brands, traditional practices, and complementary feeding. During KIIs, several prompts were used to probe about common foods (Table 1). When local names for food items were not easily translated into English, KIs were asked to share photographs, or Latin names were identified using internet searches or other published resources [[26], [27], [28]]. Terminology was often checked with photographs found on the internet during videoconferences (asking, “What do you call this?”). We aimed to identify sentinel foods for each food group that would capture >90% of the national population who consumed any item in the food group. Principles for selecting sentinel foods are listed in Table 2 [[3], [4], [11], [13], [29], [30], [31]]. Within this target, questions were kept as brief as possible, with a maximum of 7 items per question [11,13,30]. KIs were asked to rank the popularity of each food in its respective food group to help narrow down food lists and prioritize the most common items. Ranking was also used to help order items in the question, starting with the most salient items—those that are thought of first and are consumed by a high proportion of people. KIIs continued until saturation was reached: that is, until minimal new information was gained from each additional interview, which required an average of 7–8 KIIs per country. The revision and finalization process included country team feedback from the 3 international survey organizations implementing the adapted questionnaires: the GWP, DHS, and the LSMS, as well as other country team feedback where governments or research groups were implementing the DQQ in national surveys.

**TABLE 1:** Prompts during key informant interviews.

Prompt theme	Examples
Probing within each food group to consider seasonality, consumption manner, frequency, amounts, and population recognition	General: • Are there any other foods for [food group] that have not been mentioned? • Think about how people refer to each food—are there other commonly used names for any of the foods you listed in [food group]? • Are there popular dishes that use [food group/food item] as a primary ingredient that people may not always think of? • If asked, “Did you eat ___ yesterday,” and someone ate “___,” would they say yes? Seasonality: • Are there any foods common in certain seasons that are missing? How many months is the food in season? Demographic: • Are there any specific foods commonly eaten by lower, middle, and/or higher socioeconomic populations? • Are there any foods common in urban areas that would not be found in rural areas or vice versa? • Is this food consumed in all regions of the country? Are there foods we have missed that are common in specific regions of the country? Frequency: • How often is this food consumed: daily, weekly or monthly? Is it consumed only for special occasions?
Prompts once food groups are saturated	• Does this list represent the category well? Are there any important foods missing? • Are any foods uncommon that should be removed? • What are the most common foods in this list? What would be the order if you arranged the foods in order from most commonly eaten to least?

**TABLE 2:** Principles for selecting sentinel foods.

Principle	Rationale	Examples of application
We use sentinel foods (the most commonly consumed foods) for each food group.	It is not necessary to create an exhaustive list for each food group. By focusing on only the most common foods, questions can capture 90% or more of foods consumed by a population [3,29].	• We include foods consumed in different seasons (especially fruits and vegetables) to enable survey use throughout the year. • Foods may be available but unaffordable for most. A food that is more affordable is more likely to be included than a food that is less affordable. • We consider availability of foods across geographical location, urban and rural demographics, with special attention to industrially produced foods (i.e. salty snacks and soft drinks) and foraged foods (i.e. fruits).
The sentinel foods include commonly consumed foods among large subpopulations, but not small subpopulations.	The DQQ should be valid for national representation and major groups/regions of the country; minority groups who are a very small proportion of the population (eg indigenous groups in Brazil) are typically not sampled in national surveys and may have very different sentinel foods.	• We include regionally relevant foods to avoid underestimating consumption of important local foods of certain population groups. • As a rule of thumb, we include foods relevant to subpopulations if they are >10% of the total population.
Foods are categorized according to consistent category definitions.	Foods are categorized in the same way across countries, for global comparability. (For example, potatoes are always classified as a starchy root/tuber, even in countries where they are considered a vegetable.)	• The DQQ food groups are aligned with the categorization of foods in MDD-W [11]. • The IYCF DQQ food groups are aligned with the categorization of foods in WHO and UNICEF [4]. • For food groups not in these references (e.g. whole grains, baked vs. other sweets, fast food, and ultraprocessed salty snacks), we defined the category. • All food group definitions are in Supplemental Table 2 and available at dietquality.org.
We limit each question to 7 sentinel food items.	People can typically hold 7 items in their working memory [30].	• The least diverse food groups (i.e. eggs and cheese) typically only have 1–2 items that capture the category. The most diverse food groups (i.e. fruits and vegetables) may be captured through 2–3 questions to account for seasonal variety. • An additional item (8 total) may be included if there are 2 equally relevant terms for items (i.e. hot dogs or sausage).
We pay attention to the order of foods within a question.	The “primacy effect” means that people will focus more on the first thing they hear [31].	• For each question, items are ranked cognitively and appear in order of frequency, with rarest items listed last. • We list similar items together (i.e. rice, rice porridge). • Harmonization ensures that the ordering of foods follows a similar pattern across regions, improving comparability across countries.
We ask about foods and beverages using terms people understand in the country context.	Questions should be kept simple with recognizable common terms.	• Instead of asking about wheat flour or corn flour, we ask about common foods made from it (i.e. bread, tortillas, and pasta). • If pounded tuber paste is known as “fufu,” we use this term. • Brands are only used where they are a necessary reference (e.g. “Two-minute noodles” or “Indomie” are brands that in some contexts are used as generic terms meaning “instant noodles”).
We avoid names of items that may easily be confused with unrelated items.	Because of linguistic similarities between unrelated food items, it is important to use terms that clearly distinguish them or drop items that are less common and easily confused.	• For example, unless there is a specific local term, we avoid asking about “orange banana” or “tree tomato,” relatively uncommon vitamin A–rich fruits that many people do not know, because respondents may hear and answer only about the words “banana” or “tomato” (which are extremely common and in different food groups). • We are careful to disambiguate the term “beans” (pulses) and “green beans” (a vegetable).
We do not expect respondents to be able to distinguish between plant varieties.	While some respondents can distinguish between varieties, this is not an awareness common to all respondents, so asking about specific varieties is likely to result in confusion or misclassification.	• Some varieties of lettuce are vitamin A rich and fit in the dark green leafy vegetable category while others do not. In most countries/languages, respondents cannot differentiate specific varieties of lettuce, so “lettuce” is included in “other vegetables” to avoid overinflating the dark green leafy vegetable category. • Exception: we ask respondents to differentiate sweet potato and melon varieties by color, for example, in the terms “sweet potatoes that are orange inside” and “orange melon/cantaloupe.”
We use broad language where possible, avoiding excessive examples.	Umbrella terms can be useful ways to capture multiple foods in a succinct way [13].	• In most cases, we ask about fish in general instead of enumerating each species that may be consumed in a given country. • In most cases, we ask about beans in general instead of listing the different colors and varieties available. • Rice can be prepared in many different ways. It is usually sufficient to just ask about “rice” rather than list all dishes made with rice (e.g. “rice” captures “lemon rice” and “fried rice” and “pulao”).
We use specific dish names where people might not think of an important food.	We cannot assume that all consumers know the composition of common dishes.	• We include some commonly prepared foods—like tofu, hummus, and khichdi, majadra—instead of asking only about the legumes they are made with (soy, chickpeas, and lentils).
We err on the side of avoiding overreporting of the food group.	There are foods that may be common but are not a useful measure of whether an individual consumed a given food group—and analyze the risk of overinflating specific indicators.	• Onions are one of the most universally common foods around the world. While they may be consumed in large amounts, they are also commonly used in small amounts to flavor dishes. Because of their ubiquity and frequent use as a flavoring, they are not reliable for indicating vegetable consumption in populations and are therefore excluded. • In many countries, brown bread may appear to be whole grain but have very little whole grain content, so asking about “brown bread” may overinflate the category.
We include items trending toward consumption.	Being future-oriented allows the monitoring of key trends over time.	• Even if not very popular now, we include foods that are a growing export or being increasingly promoted by governments (e.g. orange-fleshed sweet potatoes in several African countries and macadamia nuts in Rwanda), and items that are increasingly marketed (e.g. energy drinks).
Exclusions		
Foods consumed in small (<15 g) amounts.	Certain foods are most commonly used in small amounts to flavor dishes, and including them may overinflate food groups.	Small amounts: • Lemon, lime • Garlic, ginger, chili peppers • Spices, herbs • Condiments • Niger seeds • Sesame seeds in countries where they are typically consumed as a sprinkle
Foods that are unnecessary to include.	Certain foods may be common but other sentinel foods are so common that the vast majority of consumers of the food group would already be covered. Some foods are already captured by other items.	Examples: • Breakfast cereal is unnecessary in most cases because it is unlikely to be the only staple food made from grain consumed in a day (DQQ group 1); people who ate cereal probably also ate already-listed sentinel foods such as bread, rice, or pasta. • Sugar and honey: already captured in sweet beverages and sweet foods.
Foods that may be considered offensive.	Due to cultural or religious practices, asking about certain foods may endanger the whole question or survey, or the enumerators.	Examples: • Where there are religious restrictions on eating meat (e.g. beef in Hindu populations and pork in Muslim populations), if pork is included as a sentinel food, participants may skip the entire question or cancel participation in the interview. Therefore, these items are excluded.
Foods that do not fit clearly into food groups.	Not all foods or beverages fit into the 29 food groups. Moreover, products with unclear or varying composition are not useful sentinel foods in indicators of diet quality.	Examples: • Plain tea and coffee • Water • Street foods • Cooking ingredients: salt, sugar, baking powder, flour, fats and oils

### Harmonization

Following an independent adaptation in each country, a process of regional harmonization served to identify anomalous inclusions or exclusions in individual countries within a region. Furthermore, sometimes insights from one country applied to others. For example, in trying to differentiate which foods were made from whole grain or refined grain, rare KIs could provide accurate technical information, and published resources might be available only for a specific country: for example, Ghanaian food blogs explain different kinds of “swallows” (staple foods) [32]. In the harmonization process, we applied information and insights from individual countries to neighboring countries (e.g. “swallows” in Togo and Benin) to ensure consistent inclusion, exclusion, and categorization of food items across borders. We compiled a global database of all semifinal sentinel foods and beverages from each individual country. Regional comparisons of all foods and beverages were then analyzed and compared using the following geographic regions: Latin America and the Caribbean, Northern Africa, Western Africa, Central Africa, Southern Africa, Eastern Africa, Middle East, Mediterranean, Commonwealth of Independent States and Central Asia, South Asia, Southeast Asia, and the Pacific. We also used maps to visualize specific foods and beverages across regions to assess consumption consistency and trends. Maps were created with online software, mapchart.net, including the figures in this article. Where regional inconsistencies were found, we followed up with KIs from each respective country to ensure consistent inclusions, exclusions, and number of brand name examples (where applicable).

Once sentinel foods were identified and harmonized, questions were finalized. In every question, we considered the order of items that makes the most sense, based on serial position effect [31] and other considerations for cognitive ease. Generally, the most common items are placed first and proceed in descending order [11], due to the psychological principles of salience and primacy—the first items in a list are better remembered than items in the middle of a list [33]. This rule was not strictly followed because we also considered the similarity of items (e.g. peaches and nectarines go next to each other, as do melon and watermelon, and dates and other dried fruit), as well as linguistic similarity of items and how the words flow off the tongue when spoken, for cognitive ease in listening to lists of items. Translations were done by the adaptation team directly for languages spoken by the team: French, Spanish, Portuguese, Russian, Armenian, and Turkish. Translations for other languages were outsourced to a translation company, directly or via Gallup. Some translations were provided by KIs.

## Results

**FIGURE 1:**
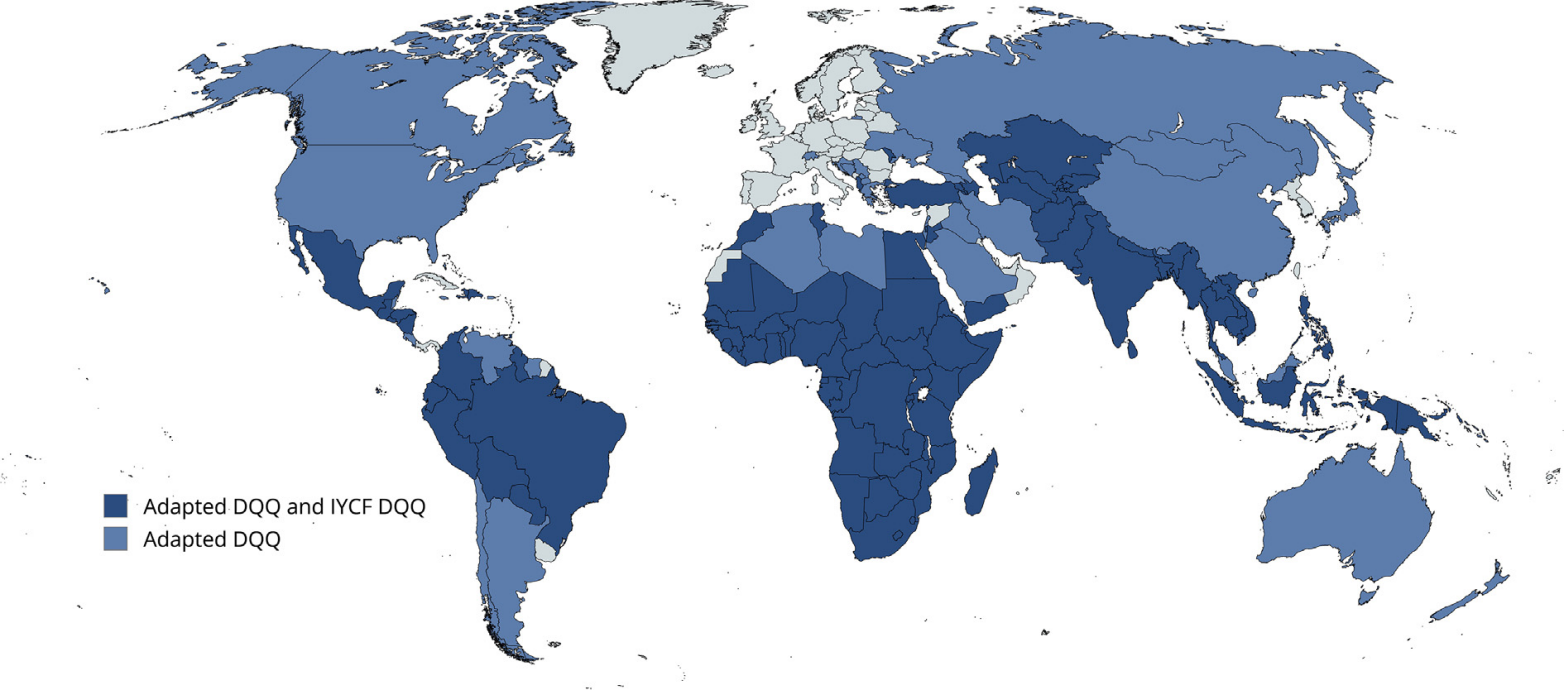
Country-adapted DQQ and IYCF DQQ available. DQQ, diet quality questionnaire; IYCF, infant and young child feeding.

Between 2020 and 2024, 6 members of the adaptation team (the authors) adapted DQQs for 140 countries with the input of a global network of >1000 KIs. IYCF DQQs were also adapted in 96 countries. Translations into other non-English languages resulted in 143 non-English questionnaires. A map of countries where adaptations have been completed is shown in Figure 1, and a list of all translated DQQs and IYCF DQQs can be found in Supplemental Table 3. All adapted and translated tools are published online at dietquality.org/tools [34]. In total, ∼9550 h were invested in the adaptation process for the DQQ and IYCF DQQ, ∼68 h per country (Table 3).

**TABLE 3:** Key informant interview summary, 2021–2024.

	*n*	%
Total key informants involved	1016	100
UN agencies	230	23
FAO	79	8
WFP	61	6
WHO	28	3
UNICEF	53	5
Other (e.g. IFAD, ILO, and UNFPA)	11	1
Government (Ministry/NSO/agency staff, and SUN focal points)	207	20
NGO	82	8
University	261	26
Others (e.g. dietitians, agronomists, food science specialists, and residents)	234	23
Average key informants per country	7–8	
Hours of interview time contributed by key informants	1524	
Hours of preparation, interviews, review, and harmonization by the adaptation team	8026	
Total hours invested in adaptation: DQQ for 140 countries + IYCF DQQ for 96 countries	9550	
Hours per country (average)	68	

Abbreviations: DQQ, diet quality questionnaire; FAO, Food and Agriculture Organization of the United Nations; IFAD, International Fund for Agricultural Development; ILO, International Labour Organization; IYCF, infant and young child feeding; NGO, non-governmental organization; NSO, national statistical office; SUN, Scaling Up Nutrition; UNFPA, United Nations Population Fund; WFP, World Food Programme; WHO, World Health Organization.

### Key informant interviews

A total of 1016 KIs contributed their local expertise to the adaptations, of which ∼23% of KIs were UN agency staff in countries, 20% government staff, 8% staff from NGOs, 26% from academia, and 23% were others including nutritionists, agriculturalists, food technologists, and independent consultants. KIs from diverse technical backgrounds and regions provided important perspectives on regional and rural/urban consumption differences, degree of processing, availability of certain items, seasonality, and socioeconomic differences of populations. On average, 7–8 interviews per country were needed to reach a level of saturation on the information needed to create the lists of sentinel foods for the DQQ. KIIs for these 140 countries took 1524 h in total (Table 3).

### Sentinel food selection

The average number of sentinel foods identified per country in the DQQ is 126, with a range of 90 (Kiribati) to 159 (Thailand). The number of items by food group is shown in Table 4. Numerous challenges arose when identifying, prioritizing, and categorizing sentinel foods, many of which required additional research and follow-up contact with KIs to clarify solutions; examples are shown in Table 5. Criteria for exclusions and excluded foods are in Supplemental Tables 4 and 5. Previously published wording for IYCF questionnaires [4], intended to be universally understood, posed problems for some contexts or languages and required adaptation; a summary of adaptation issues specific to IYCF are shown in Table 6.

**TABLE 4:** Number of sentinel foods identified by food group.

	Food group	Average (range) No. of sentinel items
1	Foods made from grains	5 (2–10)
2	Whole grains	5 (1–8)
3	White roots, tubers, and plantains	5 (1–9)
4	Legumes	5 (1–8)
5	Vitamin A–rich orange vegetables	3 (1–6)
6	Dark green leafy vegetables^1^	8 (1–14) (4–7 per question)
7	Other vegetables^1^	11 (7–19) (4–7 per question)
8	Vitamin A–rich fruits	4 (2–7)
9	Citrus	3 (0–5)
10	Other fruits^1^	13 (7–21) (4–7 per question)
11	Baked/grain-based sweets	5 (2–8)
12	Other sweets	6 (3–8)
13	Eggs	1 (1–5)
14	Cheese	2 (0–6)
15	Yogurt	2 (0–7)
16	Processed meats	5 (1–8)
17	Unprocessed red meat (ruminant)	4 (1–7)
18	Unprocessed red meat (nonruminant)	3 (0–7)
19	Poultry	3 (1–8)
20	Fish and seafood	5 (1–8)
21	Nuts and seeds	5 (2–8)
22	Packaged ultraprocessed salty snacks^2^	1 (1–3)
23	Instant noodles^2^	1 (0–4)
24	Deep fried foods	6 (1–8)
25	Fluid milk	3 (1–6)
26	Sweet tea/coffee/cocoa	4 (2–8)
27	Fruit juice and fruit drinks	4 (1–7)
28	Sugar-sweetened beverages (soft drinks, energy drinks, sports drinks)^2^	3 (1–4)
29	Fast food^2^	1 (1–1)
	All items	126 (90–159)

Data are shown for 140 countries.

1 The food group is asked in 1–3 questions that are combined in the analysis stage.

2 Brand names used to illustrate examples of the item(s) in this food group are not counted as separate items (e.g. soft drinks such as Coca-Cola, Fanta, or Sprite is 1 item).

**TABLE 5:** Sentinel food list selection challenges.

No.	Food group	Examples of challenges
1	Staple grains	In many countries, the challenge was to identify only the most commonly consumed items. In all countries, there are multiple forms in which staple grains (mainly maize, wheat, and rice) are consumed. Many of these are unnecessary to list (e.g. breakfast cereal) because a very small proportion of the population would have consumed those specialty items as the only staple grain food in a day. Substantial probing was needed for this question to identify the staples used most. Often, it was challenging to know whether multiple forms of a similar food could be combined into a single item or not. For example, in India naan or paratha had to be named separately, they are not generalized as “bread”. On the contrary, “rice” in India would capture many formulations of rice (curd rice, lemon rice, coconut rice, tomato rice, and biryani), so only one term was needed. Examples of misclassification: - Porridge from legumes or tubers - Sweet biscuits and cakes
2	Whole grains	Discerning between refined grain and whole grain foods sometimes required extensive research and consultation. Many corn-based foods (e.g. bread, porridge, tortillas, arepas, fufu) were classified according to processing methods in each country. “Brown” bread may appear to be whole grain but instead reflect additives or syrups (e.g. common in many countries in the Americas). Examples of misclassification: - Colored (brown or gray) bread - Porridges usually made from refined grain
3	White roots/tubers	Common dishes from processed roots and tubers (like gari in West Africa) are included in this list. In some countries, green, unripe banana is also consumed as a starchy staple food and is different from plantain. Examples of misclassification: - Ripe banana
4	Legumes	In Southeast Asian countries, mung beans are more common in their sprouted form, which is classified as a vegetable instead of a legume. Examples of misclassification: - Nuts - Fermented or nonfermented bean paste - Soy sauce
5	Vitamin A–rich orange vegetables	In some African countries, the terms squash (or pumpkin) are commonly used to refer to the seeds (vs. the flesh), which are a key ingredient in soups and sauces. In these cases, squash was excluded from this category. In Indonesia and southern Africa, the word for squash would be misunderstood as a common fruit drink or soft drink. Examples of misclassification: - Yellow flesh sweet potatoes that are low in carotenoids - Yellow peppers that are low in carotenoids
6	Dark green leafy vegetables (DGLVs)	In many West African countries, it was necessary to say “leaf sauce” or a similar term, which is always made with DGLV, because respondents may not always be aware of the exact vegetables in the sauce. Most countries use herbs as garnishes and do not consume them in large amounts. However, in Armenia and neighboring countries, a plate of herbs is a common fixture of any meal and consumed abundantly. Examples of misclassification: - Cabbage (green, purple) - Other pale green leaves (iceberg) - Herbs (in most contexts)
7	Other vegetables	Some vegetable names can refer to multiple parts of the plant; for example, in Laos “Sesbania” can refer to both the flower and the green leaf, which belong in separate categories (green leafy vegetables and other vegetables, respectively), so it was necessary to specify “Sesbania flower” for the other vegetables category. Examples of misclassification: - Chili peppers - Pickles and olives - Dark green leafy vegetables
8	Vitamin A–rich fruits	Not all dark orange fruits are rich in vitamin A. For example, “monkey orange” in southern Africa is dark orange yet is not rich in vitamin A rich (nor is it a citrus fruit). In some countries, passion fruit is common, but populations are more likely to consume passion fruit–flavored beverages than the actual fruit. In this case, we excluded passion fruit so as not to overinflate the indicator with respondents reporting a passion fruit–flavored beverage. Examples of misclassification: - Unripe (green) mango or papaya or hog plum (Spondias sp.) - Citrus (oranges and mandarins) - Peaches and nectarines - Yellow-fleshed bananas
9	Citrus	In Indonesia, all citrus is referred to as jeruk, presenting a challenge for including citrus consumed as fruits (i.e. oranges, mandarins, and grapefruits) and excluding citrus used for flavoring (i.e. lemons and limes). After consultation with KIs, explaining what foods should be included vs. excluded, consensus was that “buah jeruk” means citrus eaten as a fruit and does not include lemon or lime. Examples of misclassification: - Lemon
10	Other fruits	In several contexts, naming discrepancies for common fruits were discovered. In Nigeria and Caribbean countries, pear is likely to refer to an avocado, and in Nigeria, grape is likely to refer to grapefruit. In Liberia, rambutan is known as monkeynut. In Trinidad and Tobago, banana is known as fig. Examples of misclassification: - Sweetened or processed fruit products - Fruit juice - Dried coconut (used as an ingredient) - Tamarind (used as an ingredient/paste)
11	Baked grain-based sweets	Deep-fried dough (such as puff puff, beignets, and mandaazi) can have varying levels of sweetness in various African countries. We probed about whether they are usually or always sweet, whether they are rolled or dipped in sugar after frying, and whether they are consumed as savory staples (like bread) during meals (Figure 3). A common sweet in several countries in Southeast Asia is a gelatinous dessert made from rice flour and coconut. While it contains grain, like rice pudding, it was included in the “other sweets” category due to its similarity to puddings. Examples of misclassification: - Slightly sweet staple foods (bread and porridge)
12	Other sweets	Sometimes the same name refers to different food items in different countries. In Turkey, Lebanon, and other countries, the word “halwa” refers to sweet sesame and/or semolina dessert; but “halwa” is understood as hard candy in Tunisia. “Harissa” means a wheat-based dessert in Egypt (group 11), a whole wheat porridge in Armenia (group 2), and a hot pepper paste in other middle eastern countries (excluded). Examples of misclassification: - Chewing gum (often artificially sweetened; does not contribute significantly to sugar intake)
13	Eggs	For most countries, the term egg covers all varieties (i.e. chicken, duck, and quail). In Uganda, rolex (a fried egg wrapped in a chapati) and egg roll (common breaded boiled egg) were also included in this category. Examples of misclassification: - Fish eggs (roe)
14	Cheese	In Nigeria, awara (cheese) may refer to both dairy-based and soy-based cheese. In Cambodia, prahok (cheese) is a common term for a fermented-fish side dish; dairy-based cheese is not common. Examples of misclassification: - Soy-based cheese products - Processed cheese spreads (contain little or no dairy) - Cream cheese, sour cream, or other high-fat cheese products
15	Yogurt	In several Asian countries, fermented sugar-sweetened beverages containing little dairy content Yakult and Vitagen are common and often mistaken for yogurt. In Palestine, the term laban refers to yogurt, while in Egypt, laban means milk. Examples of misclassification: - Yogurt-based desserts
16	Processed meat	The processing of sausage varies considerably from country to country. In most countries, all or almost all sausage is cured, smoked, or processed with nitrates, but in some countries, there are fresh, nonprocessed sausages (e.g. merguez in Morocco or blood sausage). Examples of misclassification: - Ground meat or minimally processed meat - Chicken nuggets - Tinned fish - Deep fried meat
17	Unprocessed red meat (ruminant)	Certain dishes (like mantu beef dumplings in Afghanistan) are listed here if they are an important form of ruminant consumption that would be missed by terms like beef. Examples of misclassification: - Camel - Horse
18	Unprocessed red meat (nonruminant)	The term bushmeat was often cited as a catch-all term for animals that may have included some ruminants (types of deer) but more often were nonruminants (wild pig, rabbit, and snake). Examples of misclassification: - Venison
19	Poultry	Some countries reported frequent consumption of chicken organs (i.e. liver and gizzards)—which would not be captured by asking about chicken in general, and were thus listed separately. Examples of misclassification: - Liver or organs from other animals, if insufficiently specified
20	Fish and seafood	The term seafood is not always an effective catch-all term, especially in countries that have diverse seafood (i.e. Samoa) or those that have very little (i.e. only in canned form). In Liberia, the common term for shrimp is crabfish. Examples of misclassification: - Fish powder - Fish sauce - Fish roe - Snails - Seaweed
21	Nuts and seeds	Several countries use groundnuts in large quantities to make stews and soups. Common names for these dishes were included. This category includes nut butters, and some nut-based and seed-based desserts, for example, halwa in the Middle East or benni cake in Sierra Leone, both of which contain large amounts of sesame seeds per serving. Examples of misclassification: - Nut-based milks (nutritionally dissimilar to nuts) - Coconuts (fruit, not a nut) - Tiger nuts (starchy root, not a nut) - Kola nuts (stimulant, nonnutritive) - Bambara groundnuts (legume, not a nut)
22	Ultraprocessed packaged salty snacks	In most countries, chips is used as a catch-all term to capture ultraprocessed packaged salty snacks. In some countries where homemade chips are common, brand names like Lays and Pringles or qualifiers (i.e. packaged and store-bought) were needed to elicit the correct reference. Examples of misclassification: - Nuts - Packaged snacks that are not salty (e.g. rice crackers)
23	Instant noodles	In many countries, this product is known as “noodles”; however, to avoid confusion with pasta (which belongs in foods made from grains, category 1), the question was often formulated as “[instant] noodles such as [popular brand names like Indomie, Maggi noodles, Wai Wai or Rolton].” Examples of misclassification: - Regular noodles (e.g. pasta and spaghetti)
24	Deep-fried foods	Savory pastries (such as bourek in Middle Eastern countries) are classified here due to their high fat content, although they are not deep fried. For fried meat, we differentiate between pan-fried foods and deep-fried items (like breaded and fried chicken), even though in some cases pan-fried foods may have high amounts of oil, because the amount of oil is more variable according to individual preference when pan-frying. Examples of misclassification: - Donuts (classified as a sweet)
25	Fluid milk	Milk is not always recognized as a fresh or liquid beverage. In some countries (e.g. Solomon Islands and Papua New Guinea), only the powdered form is sold. In other countries, the term “powdered milk” is not clearly understood (e.g. Myanmar). Examples of misclassification: - Nondairy milk (soy, coconut, and nut-based) - Sweetened condensed milk - Cream and other high-fat dairy products - Milkshakes and other milk-based desserts
26	Sweetened tea/coffee/milk drinks	In some West or Central African countries, kinkeliba is a common sweetened herbal tea (included), while in other places, it is more common as a medicinal treatment (excluded).
27	Fruit juice	Fruit juice and fruit-flavored drinks are commonplace in many countries, but other nonfruit beverages like bissap (hibiscus drink), djinja (ginger drink), zoom koom (fermented millet drink), or sugar cane juice were also included here.
28	Soft drinks	In Russia and several Eastern European/Central Asian countries, lemonade is an umbrella term for carbonated soft drinks and does not necessarily refer to a lemon-flavored beverage.
29	Fast food	In high- and middle-income countries, global fast-food chains such as McDonalds or KFC were often listed. However, in low-income contexts where chains are less common, we used phrases such as “places that serve burgers, fried chicken, or pizza.” Examples of misclassification: - Street food - Chinese food

**TABLE 6:** Specific questions for adapting the DQQ for Infant and Young Child Feeding (IYCF) indicators [4].

No.	Food group	Examples of challenges
3	First 2 days after delivery	• In several countries, newborns may be given herbal mixes or other traditional concoctions. These were included but KIs in several countries suggested this to be a disappearing practice.
6b	Infant formulas	• The term “infant formula” is not always common in other languages. In some countries, the common term literally translates as “baby milk,” which is an ambiguous term in English. In these cases, we noted the translated term carefully, and back-translated the term into English as “infant formula” to be clear on the intended type of item. • Given the ambiguity of the term in some languages, brands were often listed after the local term for “infant formula,” because it helps people understand the question: “Infant formula, such as [Brand 1], [Brand 2], or [Brand 3]?” Listing brand names alone could confuse participants, as companies producing infant formula may also sell toddler milk, regular powdered milk, and other products.
6c.25	Milk	• The question from the WHO and UNICEF model questionnaire [4]: “Milk from animals, such as fresh, tinned, or powdered milk?” was not suitable for many contexts and languages. • The term milk from animals can be misunderstood by respondents in some countries. Alternatives included naming the animal (i.e. cow milk and goat milk) or saying milk of animal origin where the phrase was best understood for a given language (i.e. Spanish, French, and Portuguese). • The terms fresh and tinned do not always mean the same thing: fresh may be perceived to reference only raw, nonpasteurized milk (straight from the cow, and not packaged), while tinned may refer to tinned infant formula or sweetened condensed milk. In many cases, the term liquid was able to capture all varieties, including fresh, raw, pasteurized, shelf-stable, tinned, concentrated, and evaporated milk. The best terminology depended on how the term is conveyed in the language of respondents.
6k	Soymilk and nut milks	• In some countries, soymilk, nut milks, coconut milk, or tiger nut milk are popular dairy alternatives but not necessarily given to infants and young children (IYC). When common, they were included. • In some cases (e.g. Lao PDR), relevant brands like Lactosoy or Vitamilk were used as a more recognizable name of the item.
7.15	Yogurt and yogurt drinks	• IYCF indicators published by WHO and UNICEF [4] call for differentiating between semisolid yogurt and liquid yogurt drinks that could be given as breastmilk substitutes. In a context where both (i.e. semisolid and liquid) were common, we probed about whether yogurt would encompass all or if separate terms could be used to discern the 2. • In many countries, particularly in Africa, key informants made no distinction between semisolid and liquid yogurt. Yogurt or yogurt and grain combinations (i.e. thiakry, chakery, and bushera) can be consumed either with a spoon or drunk from a cup. • In many countries in Central Asia/Eastern Europe, numerous forms of fermented milk are consumed, which each have distinct names; neither the more solid forms nor the more liquid forms are called “yogurt”. Because each item has a distinct name, asking respondents about “yogurt as a food or as a drink” would not make sense. • Due to the lack of a distinction between semisolid and liquid yogurt in many contexts, and distinct names of fermented milk products in many others, the question formulation in the WHO and UNICEF model questionnaire [4] did not work. It was revised in partnership with DHS, as described by Namaste et al. [14].
7org	Organ meats	• Many KIs suggested adding tripe and/or intestine, which are sometimes the most common organ meats but are excluded from the category because they are not iron-rich [4]. • In some cases, specific dishes (i.e. *dinuguan*/pork blood stew in the Philippines, *mu’lak* in Palestine, or *taka tak* in Pakistan) that are made from organ meats or blood were important words beyond liver, heart, lung, etc, because they are more recognizable ways in which the organ meats are consumed.
7insect	Insects	• Wording to disambiguate the item: In Sao Tome and Principe, conch (included in the fish and seafood category) and snail are both called *búzio* but can be differentiated as *búzio de mar* (from the sea) and *búzio de terra* (from the ground), respectively. • In some contexts, insects may be well known, but rarely consumed, restricted to specific areas, or seasonal [e.g. *zompopos de mayo* (May leafcutter ants) in Guatemala].
7red	Red palm oil	• In West Africa, red palm oil is often consumed as red palm “stew” or “sauce” (e.g. *muamba, mosaka*, *esuk*, and *sauce graine),* but more commonly used as cooking oil. To capture both, this question may be worded as “Red palm oil or red palm [stew] or [sauce]?”
	Other IYC foods included	• Porridges and purées, including from cereal grains, tubers, legumes (e.g. groundnut purée and bean porridge), fruit, and blends (e.g. corn-soy blend). • Cereal mixes, including commercial brands, such as Cerelac, Nutrilon, and Bebelac. • Powders made from soy, fish, insects, and green leafy vegetables (i.e. moringa) that are used as ingredients in porridge or homemade infant food.
	IYC foods that may be excluded	• Choking hazards: Common foods such as nuts may be processed before giving to IYC, given the choking hazard they present (eg popcorn and nuts). In these cases, we asked about the most relevant form (i.e. paste and purée) to ensure the correct item for IYC in the questionnaire. • Bitter and sour items: Foods such as grapefruit or beverages such as ginger drink may be excluded if they are bitter or sour and generally not given to IYC. • Energy drinks: These are generally considered adult beverages but may be given to IYC if consumed like other soft drinks.

### Harmonization

**FIGURE 2:**
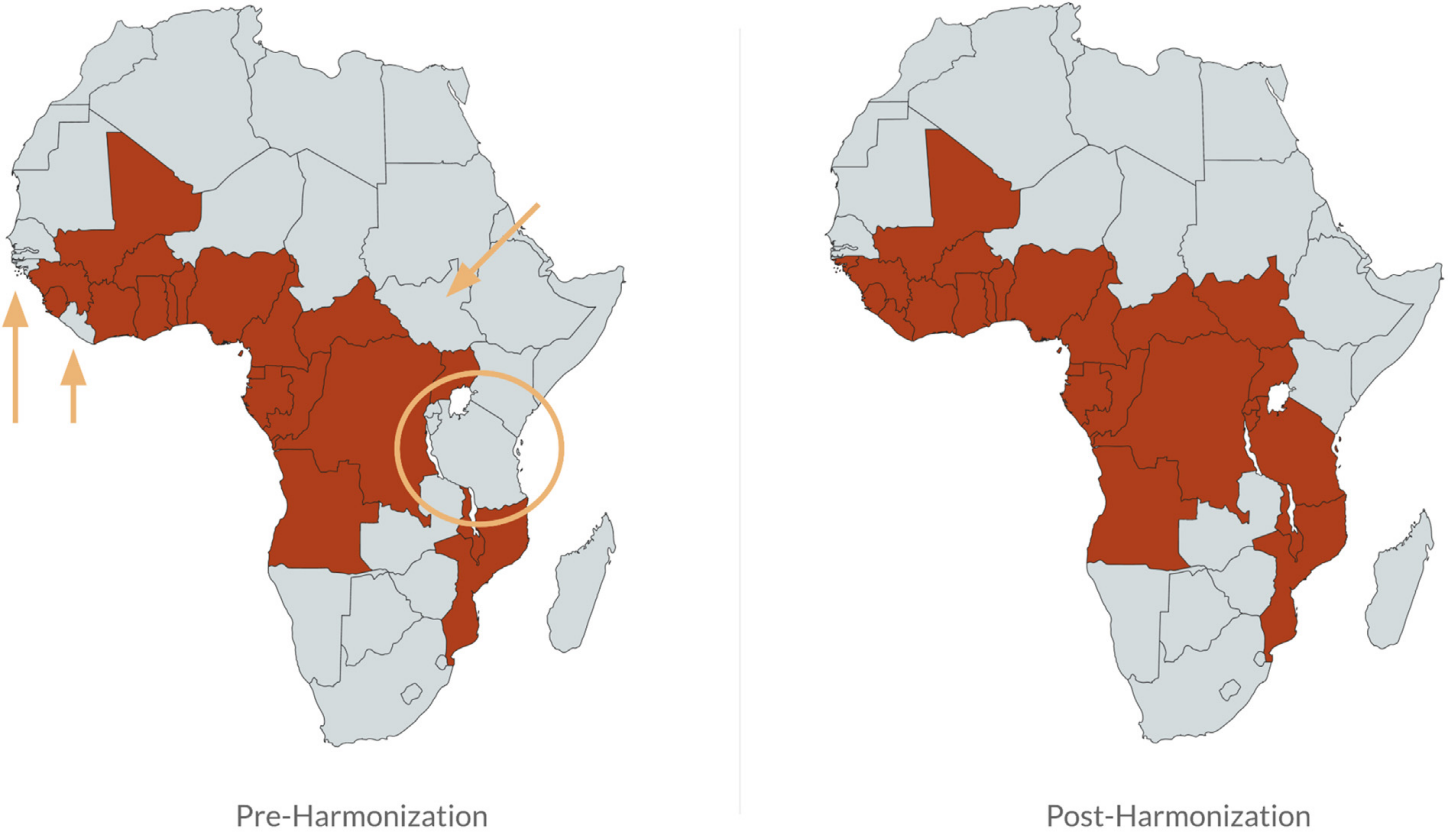
Inclusion of red palm oil in Diet Quality Questionnaires for Infant and Young Child Feeding, before and after harmonization.

**FIGURE 3:**
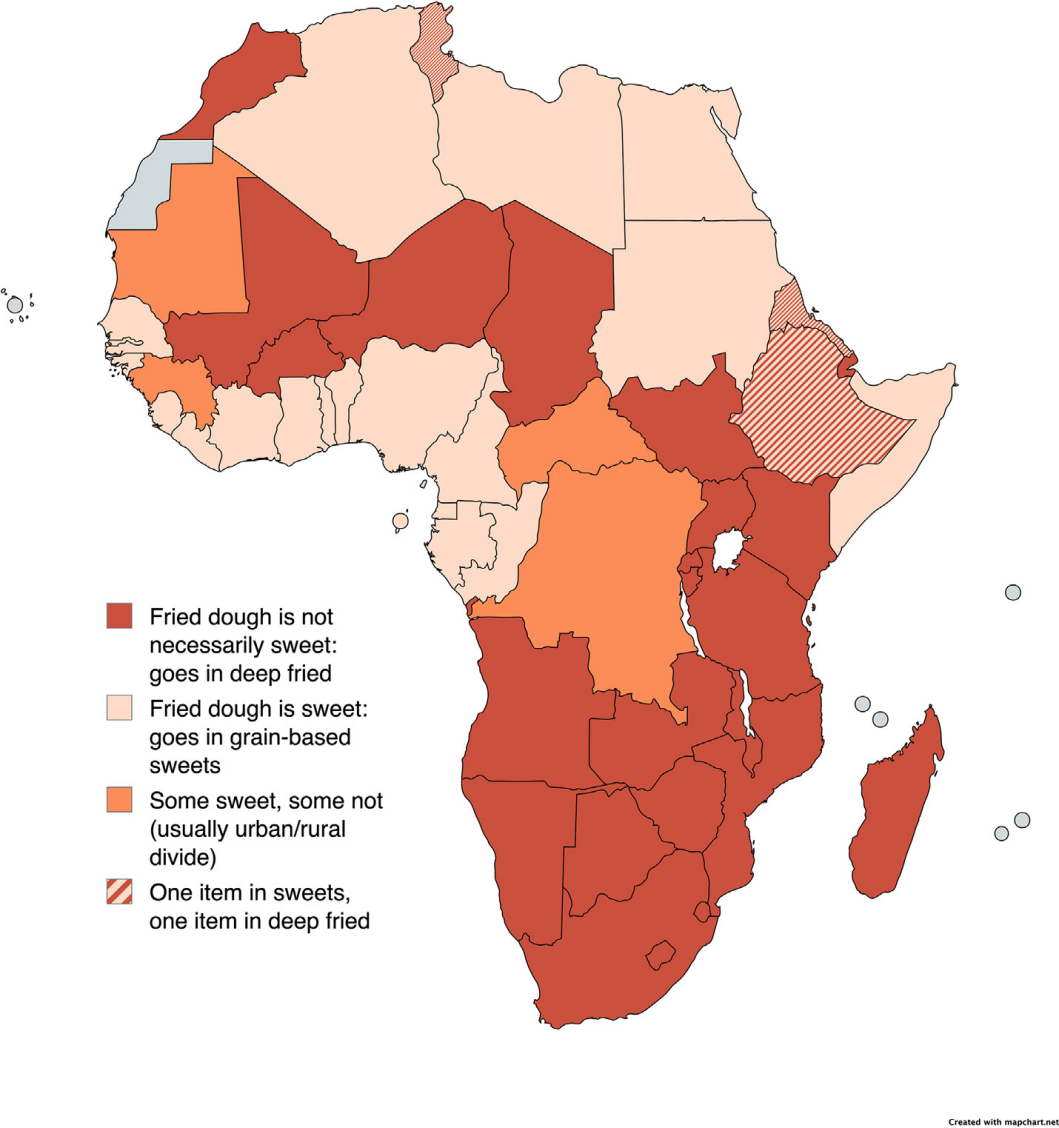
Classification of fried dough in African countries.

Reviewing the sentinel food items identified for each country, building a global database of all semifinal sentinel foods, mapping individual items across countries, and comparing items selected within regions, was the most time-intensive step of the adaptation process (Table 3). The harmonization process led to pragmatic results in terms of verifying food lists across countries and to novel insights. For example, Figure 2 shows our record of consumption of red palm oil in African countries before and after harmonization, highlighting initial omission of the item in a few countries in East and West Africa. Figure 3 shows where deep fried dough in Africa (e.g. *beignets*, *puff puff*, *waina*, and *mandaazi*) is typically sweet (categorized in the grain-based sweets food group) or not very sweet (categorized in the deep-fried foods group). These foods vary in their sweetness, some resembling a donut, and others a plain fried dough. In depth exploration of how people thought of the food (as a sweet/as a fried food), whether it was always sweet, and researching sugar content of recipes helped determine the appropriate placement of the items.

## Discussion

This research presents a global collective effort to adapt the list-based approach systematically, for food group–level data collection needed for monitoring diet quality. The list-based approach is the most feasible method for collecting food group–level dietary intake data within the DHS, GWP, LSMS, and other national surveys. Rigorous and consistent adaptations were missing, however, and the DQQ and IYCF DQQ have filled that need. The question adaptations are used for both women and children in DHS for tracking MDD-W, Minimum Dietary Diversity for children aged 6–23 mo (MDD-C), and other IYCF indicators and have been implemented in 94 countries by GWP, resulting in publicly available data [35] used for proposing MDD as an Sustainable Development Goal indicator. The DQQ is now the standard method for collecting MDD-W data in the Feed the Future Program [16] and has also been used at national level in several countries through other surveys.

The DQQs offer any survey manager the opportunity to collect data in the same way as DHS and other surveys. The availability of aligned questionnaires for both adults and children also simplifies data collection efforts across the life course. For example, a recent study about the impacts of social protection in India did not have the staff or resources to develop a diet quality survey module, but the India-adapted DQQ and IYCF DQQ were included in the evaluation, yielding novel insights about the programs’ impact on nutrition at very low marginal cost [36].

A limitation is that some countries do not yet have adapted DQQs. This study aimed to adapt DQQs for every low-income and middle-income country covered by international multitopic surveys, and exceeded that aim, including Caribbean and Pacific small island nations that are not covered by DHS or GWP alongside a few other high-income countries. Existing dietary intake survey data could provide a robust basis for adaptations in Europe, the primary missing region to date, although KIIs will still be needed to identify vernacular food item terminology.

A second limitation is that country-adapted questionnaires are not appropriate for all subnational uses. The adaptation effort sought to capture the most commonly consumed foods at the national level, including major population groups (>10% of the national population). For large subnational administrative units such as states, the DQQ is generally expected to be valid. In China, one of the largest countries with diverse regional foods, the national-level sentinel food selection was valid for subnational level; it captured over 95% of people consuming each food group in almost all provinces, for adults, adolescents, and children [29,37]. At smaller administrative levels, or for specific subpopulations, the country-adapted DQQ may miss sentinel foods important for subpopulations. Cities or districts where the population is significantly different from the national population in terms of ethnicity and/or ecology, would require specific adaptations. Adjustment for subnational populations is done by retaining the country-adapted DQQ as is, and adding separate questions containing additional items that are sentinel for the subpopulation. For example, KIs in Canada have collaborated on a DQQ adaptation for First Nations (indigenous) respondents; among other additions, in the other fruits category an additional question was added on indigenous and wild fruits. Measuring diets among immigrant populations might hybridize 2 questionnaires, one from the country of origin [13] and one from the country of residence. When extra questions for a food group are asked, the analysis would combine yes responses for the original and additional questions, and the final questionnaires should be clearly reported. We urge caution in further adaptation for subpopulations, to avoid the 5 common errors in adaptation listed earlier.

A third limitation is that the adaptations are inevitably imperfect. KIIs continued until saturation was reached, but additional information can always marginally sharpen the food list further. It is probable that the DQQ questions contain more food items than necessary in most cases and that shorter lists could gather accurate data at the national level and reduce respondent burden. However, erring on the side of longer lists buffers against the need to update questionnaires based on changes in diet patterns over time and also makes it more likely that the adapted DQQs will perform well at subnational level. The questionnaires cover on average 126 items; some items are more common than others, and there is a wide latitude for shifts to occur in popularity of items within the existing list, without adding any new items. (For example, energy drinks or brown rice consumption could be very low now and increase over time.) Periodic reviews of brand name examples may be needed, but other items are less likely to need updates. Minor updates are already being continuously incorporated each time a national survey team implements the DQQ, and provides additional clarifications on items or translations. The DQQs posted on dietquality.org reflect the most up to date versions, benefiting from the expertise of the global implementation community, including DHS, LSMS, Gallup, and other country teams and researchers; a collaborative process we envision continuing.

Despite imperfections, the DQQ adaptations are exceptionally rigorous, consistent, and harness extensive input from each country. Other than for distinct subpopulations, the adapted DQQs are not a starting point for further adaptation; they should be used as is. (An exception is the translations, which should be corrected if translation errors are found.) Common pitfalls in adaptation (described above) can diminish, rather than enhance, the validity of the questionnaire. In studies comparing the DQQ to reference methods for dietary consumption, the DQQ and IYCF DQQ performed well, providing similar food group consumption data and population indicators when compared with data collected from a multipass 24-h recall [25] and direct observation [10]. Furthermore, these adaptations are used in country-owned surveys including DHS and LSMS, and in some cases have received other official endorsement from government ministries who collaborated on the adaptation, such as in Timor-Leste and Trinidad and Tobago.

The adaptation process resulted in 2 collateral novelties. To our knowledge, the DQQ is the first questionnaire to define whole grain foods consistently globally. Whole grains are recognized as an important protective factor against NCDs, but studies in nutrition epidemiology define whole grain consumption in diverse ways even using quantitative intake data, which makes studies difficult to compare [38]. Because recipes and whole grain content of a food can differ, often without the awareness of the consumer, no food item–based classification can perfectly capture whole grain consumption. However, consistent criteria allow for tracking change in trends over time.

While the aim of the work was to produce valid country-adapted questionnaires, the process also resulted in the first systematic identification of the most commonly consumed foods in countries around the world. By mapping foods to check for inconsistencies during the harmonization process, interesting trends appeared, illustrating cultural and ecological patterns across countries (FIGURE 2, FIGURE 3). For example, no existing resource had elucidated the geography of consumption of red palm oil, which is an optional item in DHS for understanding consumption of vitamin A–rich foods (Figure 2). These insights grew into a new research output, the World Food Map, where users can search for each individual item and visualize where it is commonly consumed (worldfoodmap.org) [39].

In conclusion, the adaptation of the DQQ has been a global collaborative effort dependent upon knowledge and input of a broad and diverse network of KIs. This process revealed the nuance, detail, and simultaneous local and global awareness needed to adapt list-based questionnaires. Ready-to-use tools vastly simplify the endeavor of collecting dietary data and, thus, inform relevant policy and program action. The generosity of >1000 volunteer KIs around the world has resulted in tools available for all, reducing burdens on survey managers and making global-level, national-level, and program-level data collection possible.

## Author contributions

The authors’ responsibilities were as follows—AWH: designed the research and had primary responsibility for final content; and all authors: conducted the research, analyzed data, wrote the paper, and have read and approved the final manuscript.

## Conflict of interest

All authors report no conflicts of interest.

## Funding

Funding for the adaptations was received from the EU and BMZ through GIZ; USAID; FAO; UNICEF Lao PDR; the King Khalid Foundation of Saudi Arabia; and the Swiss Agency for Development and Cooperation (SDC). USAID staff provided comments on a draft of this article, and staff from FAO Caribbean, UNICEF Lao PDR, and the King Khalid Foundation participated as key informants in the Caribbean, Lao PDR, and Saudi Arabia, respectively. The supporting sources had no other involvement in the study design, data collection, analysis and interpretation of data, or writing of the report and have no restrictions regarding the submission of the report for publication.

## Data availability

Data described in the manuscript is publicly and freely available without restriction at dietquality.org.
