# Supplementary material for: Adaptation of the Diet Quality Questionnaire as a Global Public Good for Use in 140 Countries
Source: Curr Dev Nutr. 2024 Nov 2;9(Suppl 1):104499. doi: 10.1016/j.cdnut.2024.104499 (PMC12125687; doi:10.1016/j.cdnut.2024.104499)
Supplement: Multimedia component 1 [file mmc1.docx]

Herforth, A.W., Sokourenko, K., Gonzalez, B.C., Uyar, B.T.M., Bulungu, A.L.S., Vogliano, C. Adaptation of the Diet Quality Questionnaire as a global public good for use in 140 countries. Current Developments in Nutrition (2024), 104499.

[*https://doi.org/10.1016/j.cdnut.2024.104499*](https://doi.org/10.1016/j.cdnut.2024.104499)

## Supplementary Material

Table of Contents

[*Supplementary Material* 1](#_Toc185463895)

[Supplementary Table 1. Example of a local questionnaire adaptation, highlighting adaptation problems 2](#_Toc185463896)

[Supplementary Table 2. Food group definitions for the Diet Quality Questionnaire (DQQ) 6](#_Toc185463897)

[Supplementary Table 3. List of countries where adaptations have been completed and translated (DQQ and IYCF DQQ), and languages of translation 7](#_Toc185463898)

[Supplementary Table 4. Codes for exclusion criteria 12](#_Toc185463899)

[Supplementary Table 5. Global exclusions 12](#_Toc185463900)

## **Supplementary Table 1**. Example of a local questionnaire adaptation, highlighting adaptation problems

***Adapted for Rwanda by a local research team***

Key:

Red text for misclassified items;

Green text for omission of important sentinel items;

Purple text for terminology that would not be well understood;

Blue text for unnecessary items that could be removed.

|  | Yesterday, did you eat any of the following foods? |  |
| --- | --- | --- |
| 01 *staple foods made from grains* | Rice, sorghum, pasta, porridge, or bread, including sandwiches?  **Issues:**  Missing 2 of the most important staple foods: kawunga and chapati.  In this context, the colloquial term for pasta is “macaroni”.  Sandwiches do not need to be specified in this context. | Yes/ No |
| 02 *whole grains* | Wholegrain corn, brown rice, wholegrain bread, or oats?  **Issues:**  Missing 3 of the most important whole grain foods: Sosoma (a premixed grain porridge), sorghum porridge, millet porridge.  Whole grain corn is more clearly communicated as maize on the cob.  Wholegrain bread and oats are not common. | Yes/ No |
| 03 *white roots/tubers* | Irish potato, white flesh sweet potatoes, cassava, cooking bananas, or yam?  **Issues:**  Matoke is the common term for cooking bananas. | Yes/ No |
| 04 *legumes* | Beans, lentils, or soyabeans?  **Issues:**  Missing peas, a common item in this food group. | Yes/ No |
|  | Yesterday, did you eat any of the following vegetables? |  |
| 05 *vitamin A-rich orange veg* | Pumpkin, carrot, or orange flesh sweet potato?  **Issues:**  The standard term for “orange fleshed sweet potato” is “sweet potatoes that are orange inside” which leads to better translation. | Yes/ No |
| 06 *dark green leafy vegetables* | Amaranths, pumpkin leaves, or cassava leaves?  **Issues:**  Missing several common leafy green vegetables: Spinach, kale (sukuma wiki), bean leaves, sweet potato leaves, yam leaves.  Dodo is the common term for amaranth. | Yes/ No |
| 07 *other vegetables* | Onion, garlic, green pepper, aubergine, cabbage, tomato, cabbage, beet, courgette, or cucumber?  **Issues:**  Garlic and onion are categorically excluded (culinary ingredient for flavor, often consumed in small quantities).  Common vegetables may also include mushrooms and cauliflower.  Courgette is available but mostly consumed by the wealthy and unlikely to be a sentinel food. | Yes/ No |
|  | Yesterday, did you eat any of the following fruits? |  |
| 08 *vitamin A-rich fruits* | Papaya, mango, guava, or avocado?  **Issues:**  Guava and avocado are misclassified in this food group.  Missing passion fruit and tamarillo | Yes/ No |
| 09 *citrus* | Orange, lemon or tangerine?  **Issues:**  Lemon is categorically excluded (culinary ingredient for flavor, often consumed in small quantities) | Yes/ No |
| 10.1 *other fruits*  10.2 *other fruits* | Watermelon, passionfruit, tree tomato, or strawberry?  Dessert banana, apple, pineapple, or grape?  **Issues:**  Tree tomato is misclassified in this food group (belongs in vitamin A-rich)  Missing several common fruits: avocado, guava, jackfruit, soursop  Apples, grapes, and strawberry are uncommon and unlikely to be sentinel foods in this context. | Yes/ No |
|  | Yesterday, did you eat any of the following sweets? |  |
| 11 *baked sweets* | Cakes, cookies, sweetcakes, or sweet breads?  **Issues:**  Biscuits is a better understood term than cookies in this context. Sweet breads is not a well understood term in this context.  Sweetcakes is duplicative of “cakes” in this context and not needed. | Yes/ No |
| 12 *other sweets* | Ice cream, candy, or chocolate bars?  **Issues:**  “Candy” and “chocolate bars” are better known as “bonbons” and “chocolates” in this context. | Yes/ No |
|  | Yesterday, did you eat any of the following foods of animal origin? |  |
| 13 *eggs* | Eggs?  **No issues** | Yes/ No |
| 14 *cheese* | Cheese?  **No issues** | Yes/ No |
| 15 *yogurt* | Yogurt?  **Issues:**  Missing “ikivuguto”, fermented sour milk that is much more common than yogurt. (The term “yogurt” is understood as packaged refrigerated products sold in urban areas, and does not capture ikivuguto.). | Yes/ No |
| 16 *processed meat* | Sausages, ham, or hot dogs?  **Issues:**  Ham and hot dogs are not common in this context and can be removed. | Yes/ No |
| 17 *unprocessed red meat (ruminant)* | Beef?  **Issues:**  Missing goat, sheep, and offal, which are commonly consumed. | Yes/ No |
| 18 *unprocessed red meat (non-ruminant)* | Pork or goat?  **Issues:**  Goat is misclassified in this food group (it is a ruminant animal).  Rabbit may be important for some areas of the country. | Yes/ No |
| 19 *poultry* | Chicken? | Yes/ No |
| 20 *fish & seafood* | Fish or seafood?  **Issues:**  Missing dried fish (injanga) and sardines, which are commonly consumed and not necessarily associated with the word “fish”, which generally refers to larger fish.  Seafood is not common and not necessary to ask. | Yes/ No |
|  | Yesterday, did you eat any of the following other foods? |  |
| 21 *nuts & seeds* | Peanuts, cashews, almonds, or macadamia?  **Issues:**  Missing Gnut sauce (groundnut sauce), sunflower seeds, and pumpkin seeds.  Groundnuts is a better understood term than peanuts in this context.  Cashews and almonds are uncommon and unlikely to be sentinel foods in this context. | Yes/ No |
| 22 *ultra-processed packaged salty snacks* | Packaged salty snacks such as potato chips?  **Issues:**  The term “packaged salty snacks” should not be used because it can be misunderstood. “Crisps” is a better understood term than “potato chips” in this context, and can be used as the entire question. | Yes/ No |
| 23 *instant noodles* | Instant noodles or instant soup?  **Issues:**  The term for instant noodles in this context is “macaroni for children.”  Instant soup is not common. | Yes/ No |
| 24 *deep fried foods* | French fries, fried cassava, chapati, doughnuts, or samosa?  **Issues:**  Chapati is misclassified in this food group (belongs in starchy staple foods).  Fried fish is missing.  Missing samosa, fried sweet potato,  The term for instant noodles in this context is “macaroni for children.” “Mandaazi” is a better understood term than “doughnuts” in this context, and “chips” is a better understood term than French fries.  Fried cassava is not common. | Yes/ No |
|  | Yesterday, did you have any of the following beverages? |  |
| 25 *fluid milk* | Milk?  **Issues:**  Missing tea with milk, and powdered milk, which are common ways of drinking milk. | Yes/ No |
| 26 *sweetened tea/ coffee/ milk drinks* | Coffee with sugar, tea with sugar, or milo?  **Issues:**  “Milo” is not common in this setting, and a better term is “chocolate flavored drinks”. | Yes/ No |
| 27 *fruit juice* | Fruit juice or fruit drinks?  **Issues:**  In this context, all fruit juice and fruit drinks are simply called “juice”. | Yes/ No |
| 28 *SSBs (sodas)* | Soda or soft drinks, such as Coca Cola, orange soda, lemon soda, Sprite, tonic, or Fanta?  **Issues:**  Missing energy drinks (such as Red Bull or Energy), which are reportedly found even in rural areas.  In this context, “Fanta” is the term that means all “soft drinks, so the question phrased as “soft drinks such as…Fanta” does not make sense. | Yes/ No |
| 29 fast food restaurant | Yesterday, did you get food from any place like KFC, Mr Chips, or pizza?  **Issues:**  There is only one “Mr. Chips” restaurant so it would not be a good example of fast food; a better question formulation is “…like KFC or other places that serve fried chicken, burgers, or pizza” | Yes/No |

## **Supplementary Table 2**. Food group definitions for the Diet Quality Questionnaire (DQQ)


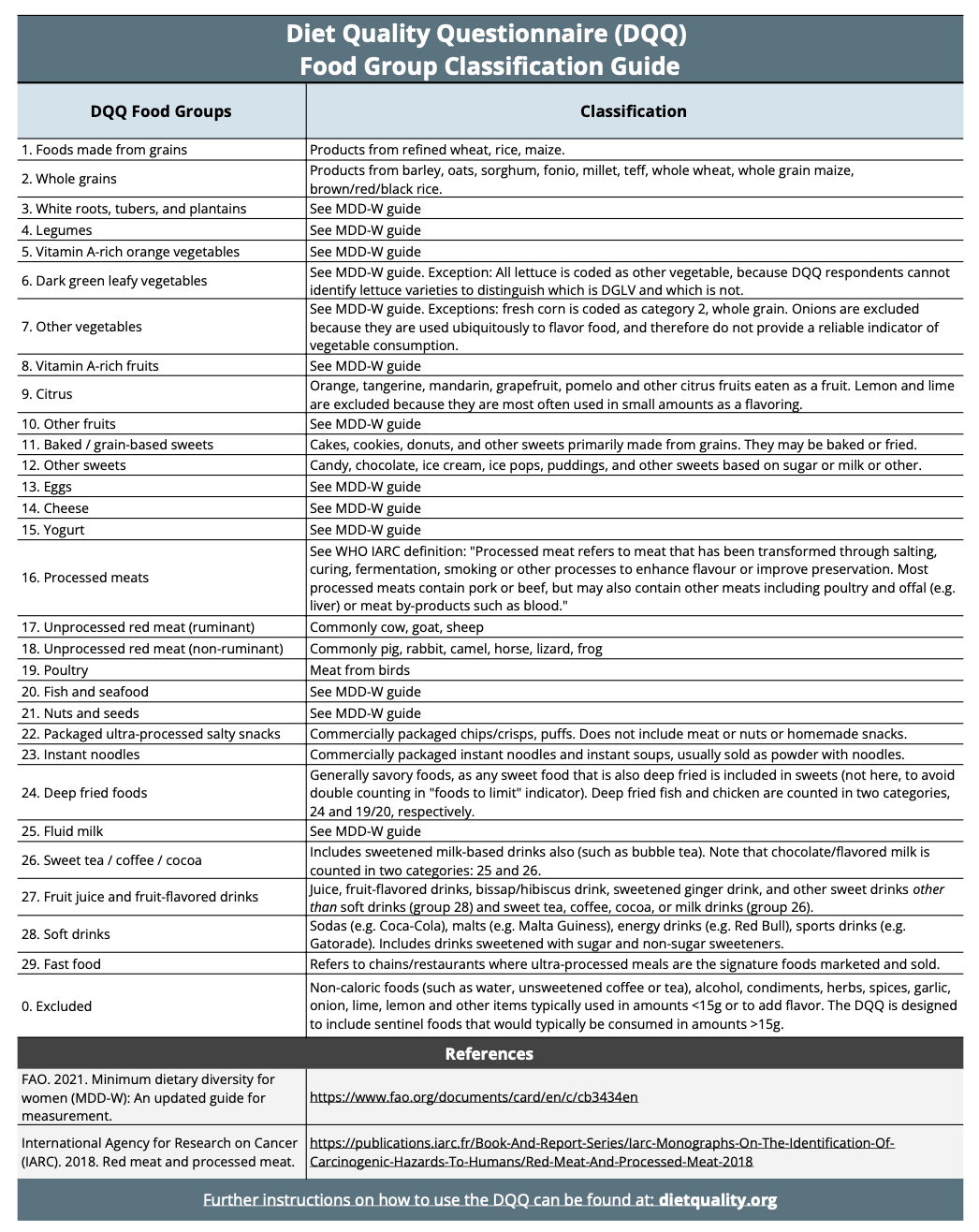


## **Supplementary Table 3.** List of countries where adaptations have been completed and translated (DQQ and IYCF DQQ), and languages of translation

| **country** | **DQQ** | **IYCF DQQ** | **number of translated versions available (non-English)** | **translations available**  **(Oct 2024)** | **Language of interview in GWP** |
| --- | --- | --- | --- | --- | --- |
| **Afghanistan** | 1 | 1 | 2 | Dari, Pashto | Dari, Pashto |
| **Albania** | 1 | 1 | 1 | Albanian | Albanian |
| **Algeria** | 1 |  | 1 | Arabic | Arabic |
| **Angola** | 1 | 1 | 1 | Portuguese | Portuguese |
| **Antigua and Barbuda** | 1 |  |  |  | n/a |
| **Argentina** | 1 |  | 1 | Spanish | Spanish |
| **Armenia** | 1 | 1 | 1 | Armenian | Armenian |
| **Australia** | 1 |  |  | English | English |
| **Azerbaijan** | 1 | 1 | 2 | Azeri, Russian | Azeri, Russian |
| **Bahamas** | 1 |  |  |  | n/a |
| **Bangladesh** | 1 | 1 | 1 | Bengali | Bengali |
| **Barbados** | 1 |  |  |  | n/a |
| **Belize** | 1 |  |  |  | n/a |
| **Benin** | 1 | 1 | 2 | Fon, French | Bariba, Fon, French, Anago |
| **Bhutan** | 1 |  |  |  | n/a |
| **Bolivia** | 1 | 1 | 1 | Spanish | Spanish |
| **Bosnia and Herzegovina** | 1 |  |  |  | Bosnian, Croatian, Serbian |
| **Botswana** | 1 | 1 | 2 | English, Setswana | English, Setswana |
| **Brazil** | 1 | 1 | 1 | Portuguese | Portuguese |
| **Burkina Faso** | 1 | 1 | 2 | French, Moore/Mossi | Dioula, French, Fulfulde, Moore |
| **Burundi** | 1 | 1 | 2 | French, Kirundi | French, Kirundi |
| **Cambodia** | 1 | 1 | 1 | Khmer | Khmer |
| **Cameroon** | 1 | 1 | 1 | English, French | French, English, Fulfulde |
| **Canada** | 1 |  | 1 | English, French | English, French |
| **Cape Verde** | 1 | 1 | 2 | Kriolu, Portuguese | Kriolu, Portuguese |
| **Central African Republic** | 1 | 1 | 1 | French | French |
| **Chad** | 1 | 1 | 2 | French, Ngambaye | French, Chadian Arabic, Ngambaye |
| **Chile** | 1 |  | 1 | Spanish | Spanish |
| **China** | 1 |  | 1 | Chinese | Chinese |
| **Colombia** | 1 | 1 | 1 | Spanish | Spanish |
| **Comoros** | 1 | 1 | 1 | French | French, Comorian |
| **Congo Brazzaville** | 1 | 1 | 2 | French, Kituba | French, Kituba, Lingala |
| **Costa Rica** | 1 |  | 1 | Spanish | Spanish |
| **Croatia** | 1 |  |  |  | Croatian |
| **Djibouti** | 1 | 1 | 1 | French | French |
| **Domenica** | 1 |  |  |  | n/a |
| **Dominican Republic** | 1 | 1 | 1 | Spanish | Spanish |
| **DRC** | 1 | 1 | 2 | French, Swahili | French, Swahili |
| **Ecuador** | 1 | 1 | 1 | Spanish | Spanish |
| **Egypt** | 1 | 1 | 1 | Arabic | Arabic |
| **El Salvador** | 1 | 1 | 1 | Spanish | Spanish |
| **Equatorial Guinea** | 1 | 1 | 1 | Spanish | Spanish |
| **Eritrea** | 1 | 1 | 1 | Tigrinya | Tigrinya |
| **Eswatini** | 1 | 1 | 2 | Siswati, English | Siswati, English |
| **Ethiopia** | 1 | 1 | 2 | Amharic, English, Oromo/Oromiffa | Amharic, English, Oromo, Tigrinya |
| **Fiji** | 1 |  |  | English | n/a |
| **Gabon** | 1 | 1 | 1 | French | French, Fang, Teke |
| **Gambia** | 1 | 1 |  | English | English, Pulaar, Wolof, Malinke |
| **Georgia** | 1 |  | 2 | Georgian, Russian | Georgian, Russian |
| **Ghana** | 1 | 1 | 1 | English, Twi | English, Ewe, Twi, Dagbani |
| **Greece** | 1 |  | 1 | Greek | Greek |
| **Grenada** | 1 |  |  |  | n/a |
| **Guatemala** | 1 | 1 | 1 | Spanish | Spanish |
| **Guinea** | 1 | 1 | 2 | French, Pular | French, Malinke, Pular, Soussou |
| **Guinea Bissau** | 1 | 1 | 1 | Kriolu | Kriolu |
| **Guyana** | 1 | 1 | 1 | Guyanese Creole | Guyanese Creole |
| **Haiti** | 1 | 1 | 1 | Creole | Creole |
| **Honduras** | 1 | 1 | 1 | Spanish | Spanish |
| **India** | 1 | 1 | 1 | English, Hindi | Assamese, Bengali, Gujarati, Hindi, Kannada, Malayalam, Marathi, Odia, Punjabi, Tamil, Telugu |
| **Indonesia** | 1 | 1 | 1 | Bahasa Indonesia | Bahasa Indonesia |
| **Iran** | 1 |  | 1 | Farsi | Farsi |
| **Iraq** | 1 |  |  |  | Arabic, Kurdish |
| **Israel** | 1 |  | 2 | Hebrew, Russian | Hebrew, Russian, Arabic |
| **Ivory Coast** | 1 | 1 | 1 | French | French, Dioula |
| **Jamaica** | 1 |  |  |  |  |
| **Japan** | 1 |  | 1 | Japanese | Japanese |
| **Jordan** | 1 | 1 | 1 | Arabic | Arabic |
| **Kazakhstan** | 1 | 1 | 2 | Russian, Kazakh | Russian, Kazakh |
| **Kenya** | 1 | 1 | 1 | English, Swahili | English, Swahili |
| **Kiribati** | 1 |  |  | English | n/a |
| **Kosovo** | 1 |  |  |  | Albanian, Serbian |
| **Kyrgyz Republic** | 1 | 1 | 2 | Kyrgyz, Russian | Kyrgyz, Russian, Uzbek |
| **Lao PDR** | 1 | 1 | 1 | Lao | Lao |
| **Lebanon** | 1 |  | 1 | Arabic | Arabic |
| **Lesotho** | 1 | 1 | 1 | Sesotho | Sesotho |
| **Liberia** | 1 | 1 | 1 | English, Kolokwa | English, Kolokwa |
| **Libya** | 1 |  |  |  | Arabic |
| **Madagascar** | 1 | 1 | 1 | Malagasy | French, Malagasy |
| **Malawi** | 1 | 1 | 2 | Chichewa, English, Tumbuka | Chichewa, English, Tumbuka |
| **Malaysia** | 1 |  | 1 | Bahasa Malay, English | Bahasa Malay, Chinese, English |
| **Maldives** | 1 | 1 | 1 | Dhiveni | Dhiveni |
| **Mali** | 1 | 1 | 1 | French | French, Bambara |
| **Mauritania** | 1 | 1 | 2 | French, Hassanya | French, Poulaar, Wolof, Hassanya, Soninké |
| **Mexico** | 1 | 1 | 1 | Spanish | Spanish |
| **Moldova** | 1 | 1 | 2 | Moldavian, Russian | Moldavian, Russian |
| **Mongolia** | 1 |  |  |  | Mongolian |
| **Morocco** | 1 | 1 | 2 | French, Moroccan Arabic | Moroccan Arabic |
| **Mozambique** | 1 | 1 | 2 | Portuguese, Xichangana | Portuguese, Xichangana, Emakhuwa |
| **Myanmar** | 1 | 1 | 1 | Burmese | Burmese |
| **Namibia** | 1 | 1 | 1 | English, Oshivambo | English, Oshivambo, Afrikaans, Kwangali |
| **Nepal** | 1 | 1 | 1 | Nepali | Nepali |
| **New Zealand** | 1 |  |  | English | English |
| **Nicaragua** | 1 | 1 | 1 | Spanish | Spanish |
| **Niger** | 1 | 1 | 2 | French, Hausa | French, Hausa, Zarma |
| **Nigeria** | 1 | 1 | 1 | English, Hausa | English, Hausa, Igbo, Pidgen English, Yoruba |
| **North Macedonia** | 1 |  |  |  | Macedonian, Albanian |
| **Pakistan** | 1 | 1 | 1 | Urdu | Urdu |
| **Palestinian Territories** | 1 | 1 | 1 | Arabic | Arabic |
| **Papua New Guinea** | 1 | 1 | 1 | Tok Pisin | n/a |
| **Paraguay** | 1 | 1 | 1 | Spanish | Spanish, Jepora |
| **Peru** | 1 | 1 | 1 | Spanish | Spanish |
| **Philippines** | 1 | 1 | 1 | Filipino | Filipino, Iluko, Hiligaynon, Cebuano, Maranao, Waray, Sorsoganon |
| **Puerto Rico** | 1 |  | 1 | Spanish | n/a |
| **Russia** | 1 |  | 1 | Russian | Russian |
| **Rwanda** | 1 | 1 | 1 | English, Kinyarwanda | English, Kinyarwanda |
| **Samoa** | 1 | 1 | 1 | Samoan | n/a |
| **Sao Tome and Principe** | 1 | 1 | 1 | Portuguese | n/a |
| **Saudi Arabia** | 1 |  |  |  | Arabic, Urdu |
| **Senegal** | 1 | 1 | 2 | French, Wolof | French, Wolof |
| **Serbia** | 1 |  | 1 | Serbian | Serbian |
| **Sierra Leone** | 1 | 1 | 1 | English, Krio | English, Krio, Mende |
| **Solomon Islands** | 1 |  |  | English | n/a |
| **Somalia** | 1 | 1 | 1 | Somali | Somali |
| **South Africa** | 1 | 1 | 1 | Afrikaans, English | Afrikaans, English, Sotho, Xhosa, Zulu |
| **South Sudan** | 1 | 1 | 1 | Juba | Juba |
| **Sri Lanka** | 1 | 1 | 1 | Sinhala | Sinhala, Tamil |
| **St. Kitts and Nevis** | 1 |  |  |  | n/a |
| **St. Lucia** | 1 |  |  |  | n/a |
| **St. Vincent and the Grenadines** | 1 |  |  |  | n/a |
| **Sudan** | 1 | 1 | 1 | Arabic | Arabic |
| **Suriname** | 1 |  |  |  | n/a |
| **Switzerland** | 1 |  | 3 | German, French, Italian | German, French, Italian |
| **Tajikistan** | 1 | 1 | 1 | Tajik | Tajik |
| **Tanzania** | 1 | 1 | 1 | English, Swahili | English, Swahili |
| **Thailand** | 1 | 1 | 1 | Thai | Thai |
| **Timor-Leste** | 1 | 1 | 1 | Tetum | Tetum |
| **Togo** | 1 | 1 | 1 | French | French, Ewe, Kabiye |
| **Trinidad and Tobago** | 1 | 1 |  | English | n/a |
| **Tunisia** | 1 | 1 | 2 | Arabic, French | Arabic |
| **Turkey** | 1 | 1 | 1 | Turkish | Turkish |
| **Turkmenistan** | 1 | 1 | 2 | Turkmen, Russian | Turkmen, Russian |
| **Uganda** | 1 | 1 | 1 | English, Luganda | Ateso, English, Luganda, Runyankole |
| **Ukraine** | 1 | 1 | 2 | Russian, Ukrainian | Russian, Ukrainian |
| **United States of America** | 1 |  | 1 | English, Spanish | English, Spanish |
| **Uzbekistan** | 1 | 1 | 2 | Uzbek, Russian | Uzbek, Russian |
| **Venezuela** | 1 |  | 1 | Spanish | Spanish |
| **Viet Nam** | 1 | 1 | 1 | Vietnamese | Vietnamese |
| **Yemen** | 1 | 1 | 1 | Arabic | Arabic |
| **Zambia** | 1 | 1 | 1 | Bemba, English | Bemba, English, Lozi, Nyanja, Tonga |
| **Zimbabwe** | 1 | 1 | 1 | English, Shona | English, Shona, Ndebele |
| **TOTAL** | **140** | **96** | **143** |  |  |

## **Supplementary Table 4.** Codes for exclusion criteria

| **Category** | **Code** | **Criterion** |
| --- | --- | --- |
| Not necessary | 1a | Not common |
|  | 1b | Least common; too many items, lowest rank. |
|  | 1c | Not sentinel; may be common, but the other items in the question already cover almost everyone who would consume the food group |
|  | 1d | Already captured by another food / grouped with another food |
|  | 1e | Anomalous: Excluded during regional harmonization |
| Excluded based on standard criteria rules | 2a | Does not belong in category (note: it may or may not be a sentinel food in the category where it belongs) |
|  | 2b | Typically consumed in small amounts (Used as a condiment, herb or for flavor) |
|  | 2c | Dubious fit in the food category (e.g. Fruit consumed primarily as juice / as preserves or as a dessert; foods that may or may not be whole grain) |
| Could be confusing | 3a | Overlaps with more than one category (e.g. “kebab” could be chicken or beef) |
|  | 3b | The terminology is confusing or not universally understood (e.g. “Tree tomato” is not widely known and easily confused with “tomato”) |

## **Supplementary Table 5.** Global exclusions

| **Q** | **Category** | **Excluded food** | **Reason for Exclusion** |
| --- | --- | --- | --- |
| N/A | none | Condiments and culinary ingredients (salt, sugar, fats, oils) | 1,2 |
| Q1 | Foods made from grains | breakfast cereal (with some exceptions) | 1c |
|  |  | crackers or savory biscuits | 1c |
|  |  | pancakes | 1c |
| Q2 | Whole grains | brown bread, unless whole grain content is universal | 2c |
|  |  | wheat germ | 1a, 2c |
| Q3 | White roots, tubers, and plantains | tapioca, unless used as a staple food | 2c |
| Q4 | Legumes | green beans  groundnuts | 2a |
| Q5 | Vitamin A-rich orange vegetables | hot pepper / chili pepper | 2b |
| Q6 | Dark green leafy vegetables | fresh or dried herbs (e.g. cilantro, parsley, basil, chives, tarragon, mint, dill, scallions, oregano) | 2b |
|  |  | lettuce | 3a |
| Q7 | Other vegetables | pickles | 2a  Excluded from the vegetables category in the MDD-W guide (FAO 2021) |
|  |  | garlic or ginger | 2b |
|  |  | onions or shallots | 2b |
| Q8 | Vitamin A-rich fruits | red palm fruit / oil palm | 2a |
| Q9 | Citrus | lemons or limes | 2b |
| Q10 | Other fruits | sugar cane | 2a |
|  |  | fruit juice | 2c |
|  |  | sweet fruit desserts (e.g. pie) | 2c |
| Q11 | Baked / grain-based sweets | specific types of cakes or cookies | 1d |
| Q12 | Other sweets | honey, sugar | 1d |
|  |  | fruit jam, jellies, conserves or marmalade | 2b / 2c |
|  |  | chewing gum | 2b / 2c |
| Q13 | Eggs | fish eggs / roe / caviar | 2a |
| Q14 | Cheese | cream cheese | 2a / 2c  primarily fat |
| Q15 | Yogurt | sour cream / creme fraiche / kaymak / ashta / sarshir / crema / clotted cream | 2a  fat/oil |
|  |  | watery probiotic drinks | 2c |
| Q16 | Processed meats | ultraprocessed meat products that are not cured, do not meet the IARC definition of processed meats (e.g. chicken nuggets, fish sticks) | 2a |
| Q17 | Unprocessed red meat (ruminant) | feet, if used as flavor | 2b |
|  |  | kebab | 3a |
| Q18 | Unprocessed red meat (non-ruminant) | feet, if used as flavor | 2b |
|  |  | kebab | 3a |
| Q19 | Poultry | feet, if used as flavor | 2b |
| Q20 | Fish and seafood | fish eggs / roe / caviar | 2a |
|  |  | snails / sea snail / snail eggs / periwinkle | 2a |
| Q21 | Nuts and seeds | small seeds, if typically consumed in small amounts as an ingredient (sesame seeds, flax seeds, chia seeds, Niger seeds, pine nuts, poppy seeds) | 2b |
| Q22 | Packaged ultra-processed salty snacks | crackers | 2c |
| Q23 | Instant noodles | plain bean threads or small rice noodles that cook quickly | 2a |
| Q24 | Deep fried foods | sweet fried foods (donuts, beignets, fritters) | 2a  (the category is savory / salty foods) |
| Q25 | Fluid milk | sweetened condensed milk | 2a |
| Q26 | Sweet tea / coffee / cocoa | unsweetened tea, coffee, or cocoa | 2a |
| Q27 | Fruit juice and fruit drinks | lemon juice used as a culinary ingredient | 2b |
| Q28 | Soft drinks | sparkling water, tonic water | 2a |
| Q29 | Fast food | street food, Chinese food, other food that is prepared fast but not necessarily ultraprocessed, coffee shops (primarily serve beverages, not meals) | 2a |
